# Oyster Reefs as Natural Breakwaters Mitigate Shoreline Loss and Facilitate Fisheries

**DOI:** 10.1371/journal.pone.0022396

**Published:** 2011-08-05

**Authors:** Steven B. Scyphers, Sean P. Powers, Kenneth L. Heck, Dorothy Byron

**Affiliations:** 1 Department of Marine Sciences, University of South Alabama, Mobile, Alabama, United States of America; 2 Dauphin Island Sea Lab, Dauphin Island, Alabama, United States of America; Institute of Marine Research, Norway

## Abstract

Shorelines at the interface of marine, estuarine and terrestrial biomes are among the most degraded and threatened habitats in the coastal zone because of their sensitivity to sea level rise, storms and increased human utilization. Previous efforts to protect shorelines have largely involved constructing bulkheads and seawalls which can detrimentally affect nearshore habitats. Recently, efforts have shifted towards “living shoreline” approaches that include biogenic breakwater reefs. Our study experimentally tested the efficacy of breakwater reefs constructed of oyster shell for protecting eroding coastal shorelines and their effect on nearshore fish and shellfish communities. Along two different stretches of eroding shoreline, we created replicated pairs of subtidal breakwater reefs and established unaltered reference areas as controls. At both sites we measured shoreline and bathymetric change and quantified oyster recruitment, fish and mobile macro-invertebrate abundances. Breakwater reef treatments mitigated shoreline retreat by more than 40% at one site, but overall vegetation retreat and erosion rates were high across all treatments and at both sites. Oyster settlement and subsequent survival were observed at both sites, with mean adult densities reaching more than eighty oysters m^−2^ at one site. We found the corridor between intertidal marsh and oyster reef breakwaters supported higher abundances and different communities of fishes than control plots without oyster reef habitat. Among the fishes and mobile invertebrates that appeared to be strongly enhanced were several economically-important species. Blue crabs (*Callinectes sapidus*) were the most clearly enhanced (+297%) by the presence of breakwater reefs, while red drum (*Sciaenops ocellatus*) (+108%), spotted seatrout (*Cynoscion nebulosus*) (+88%) and flounder (*Paralichthys* sp.) (+79%) also benefited. Although the vertical relief of the breakwater reefs was reduced over the course of our study and this compromised the shoreline protection capacity, the observed habitat value demonstrates ecological justification for future, more robust shoreline protection projects.

## Introduction

Nearshore, biogenic habitats of estuaries support a broad spectrum of marine life and serve as nursery grounds for economically-important fishes and shellfish [1]–[4]. Estuarine and vegetated nearshore habitats comprise only 0.7% of global biomes, yet the value of their ecosystem services has been estimated at $7.9 trillion dollars annually, or 23.7% of total global ecosystem services [5]. Nearshore ecosystem services include disturbance resistance, nutrient cycling, habitat, food production, and recreation. Unfortunately, coastal and estuarine shorelines are among the most degraded and threatened habitats in the world because of their sensitivity to sea level rise, storms and increased utilization by man [6], [7]. Many previous efforts to protect shorelines have involved the introduction of hardened structures, such as seawalls, rocks or bulkheads to dampen or reflect wave energy [8]–[10]. Although such structures may adequately mitigate shoreline retreat, the ecological damages that result from their presence can be great [8], [10], [11]. The cumulative effects of habitat alteration and losses in the nearshore have had substantial economic and ecological consequences [12], [13] and threaten the sustainability of many ecosystem services. Efforts to combat degradation and loss of nearshore, biogenic habitats have increased over the last decade [7], [14], [15]. Unfortunately, many shoreline protection approaches still value engineering over ecology in determining mitigation and restoration efficacy.

The “engineering first” approaches, including vertical bulkheads, concrete and granite rip-rap revetments and seawalls, are often used by coastal engineers because they are viewed as permanent and non-retreating structures. Unfortunately, insufficient concern may have been given to the ecological, aesthetic or socioeconomic impacts of these hardened structures. A major concern in implementing bulkheads and seawalls for coastal property protection is that erosive wave energies are reflected back into the water body, instead of being absorbed or dampened [10]. This subjects adjacent shorelines to even greater wave energy and can cause vertical erosion down the barrier with subsequent loss of intertidal habitats [10], [16].

The benthic setting adjacent to many armored shores is generally absent of complex, structured habitats [16]. Most structurally complex, natural habitats are thought to function as nurseries for many finfish and shellfish species because of their elevated faunal densities, enhanced growth or survival rates, or higher contribution of individuals that emigrate offshore to adult habitats [1], [4]. Biogenic, three-dimensional structure can reduce water velocities, increase sedimentation rates and enhance propagule settlement and retention, indirectly creating a more suitable environment for many species [17]–[20]. Despite the known lack of ecological benefits, shoreline hardening has continued to increase for decades primarily due to a lack of practical and ecologically valuable alternatives. However, a growing initiative for sustainable shoreline protection has focused on balancing effective protection and habitat creation by a variety of new methodologies collectively termed “living shorelines” [8].

Living shoreline projects often involve the planting or restoration of naturally-occurring biogenic habitats that have numerous ecological benefits, in addition to providing a buffer for wave action. In their natural setting, oyster reefs are often found seaward of salt marshes and can attenuate erosive wave energies, stabilize sediments and reduce marsh retreat, thereby making them an attractive living shoreline approach [19], [21], [22]. Beyond the targeted shoreline protection, living oyster reefs may provide many ecosystem services including seston filtration, benthic-pelagic coupling, refuge from predation and abundant prey resources [2], [18]. Given adequate recruitment and survival, oyster reefs could be self-sustaining elements of coastal protection [21], [22] that enhance other habitats of the natural landscape, although few studies have examined the premise of restoration through facilitation [17], [23].

**Figure 1:**
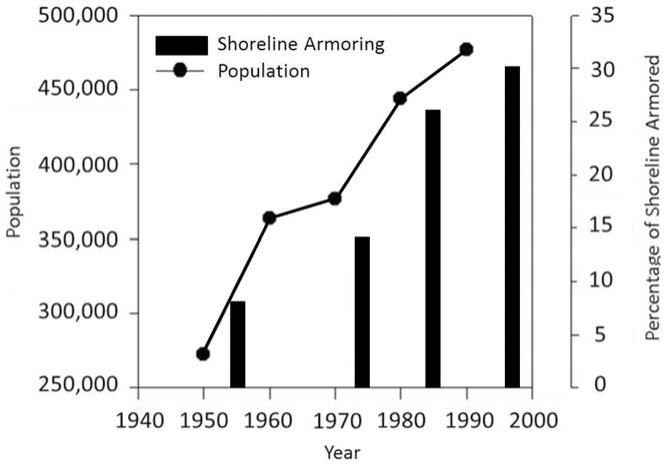
Population Growth and Shoreline Armoring in Mobile Bay, Alabama. Adapted with permission from Douglass and Pickel 1999, this figure depicts the rate and extent of shoreline armoring in Mobile Bay. The vertical bars in the main graph show the proportion of armoring while the line depicts the increasing population levels for Mobile and Baldwin Counties.

Located on the northern Gulf of Mexico, Mobile Bay is one of the best examples of a classic estuary [3] and, like many other coastal areas, is highly developed with a large and increasing proportion of its shorelines armored by bulkheads and seawalls [10] (Figure 1). At last analysis in 1997, Douglass and Pickel estimated that over 30% of the bay's available coastline was armored with over 10–20 acres of intertidal habitat lost, a high percentage in this microtidal bay (<0.5 m tidal amplitude). The historical armoring and marsh-edge losses have already had negative fisheries consequences, with projections of further reductions of blue crab harvest if armoring continues [24].

In this study, we experimentally examined the ecological effects of constructing subtidal breakwater oyster reefs for coastal and estuarine shoreline protection. In addition to documenting changes in the physical setting near breakwaters and unaltered control treatments, we quantified the habitat value for oysters, fishes and mobile invertebrates. We focus particular attention on the potential impacts on economically-important species, as this provides insight into the economic implications of different shoreline protection alternatives. We hypothesized that the addition of breakwater reefs of oyster shell would: 1) mitigate shoreline retreat, (2) provide substrate for recruitment and survival of oysters, (3) support higher densities of small fishes, mobile macro-invertebrates and larger and transient fishes and (4) promote higher species richness and a different community structure than unaltered control areas.

## Methods

### Ethics Statement

This study was conducted in accordance with the laws of the State of Alabama and under IACUC protocols (Permit # 05047-FSH) approved by the University of South Alabama.

### Study Setting and Site Selection

**Figure 2:**
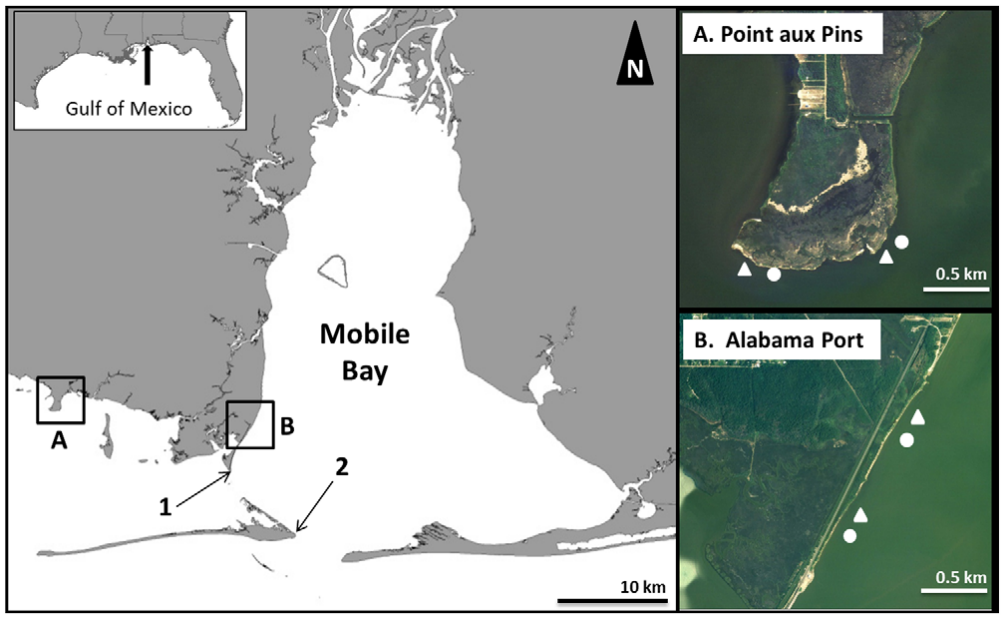
Map of Study Sites in Mobile Bay and Mississippi Sound, Alabama. White triangles represent breakwater reef complexes and white circles represent control treatments at the two restoration sites of (A) Point aux Pins and (B) Alabama Port. The locations of the (1) Cedar Point and (2) Dauphin Island hydrographic monitoring stations are denoted by the numbered arrows.

To determine the ecological and physical effects of created breakwater oyster reefs, we conducted a manipulative field experiment at two sites in coastal Alabama that contained stretches of rapidly eroding coastlines. Study sites were selected within regions known to have adequate larval supply of oysters [25] and moderate wave climates [26]. At each site, we constructed two breakwater reefs of loose oyster shell and designated non-restored plots as controls in a randomized, paired design (Figure 2). The first site, known locally as Point aux Pins, received breakwater reefs in May 2007. The treatments at Point aux Pins (site center point: 30.370098,−88.308578) were located along the southern extent of a peninsula of eroding salt marsh habitat, largely comprised of fringing cordgrass (*Spartina alterniflora*) and black needlerush (*Juncus roemerianus*). Remnants of oysters (*Crassostrea virginica*) are found throughout the marsh and buried in the subtidal sediments. The second site, Alabama Port (site center point: 30.347917,−88.121338), is located along the southwestern shore of Mobile Bay, just north of the Dauphin Island bridge. The treatments at Alabama Port were located along a two kilometer stretch of eroding shoreline that has been encroached by armoring at its northern and southern extents. Small patches of *Spartina alterniflora* can be found at Alabama Port, but the most abundant vegetation is *Phragmites* sp., which is largely present in the upper intertidal zone. Both sites were selected within regions of high oyster spat settlement (40–180 spat m^−2^ day^−1^) [25].

### Breakwater Reef Dimensions

**Figure 3:**
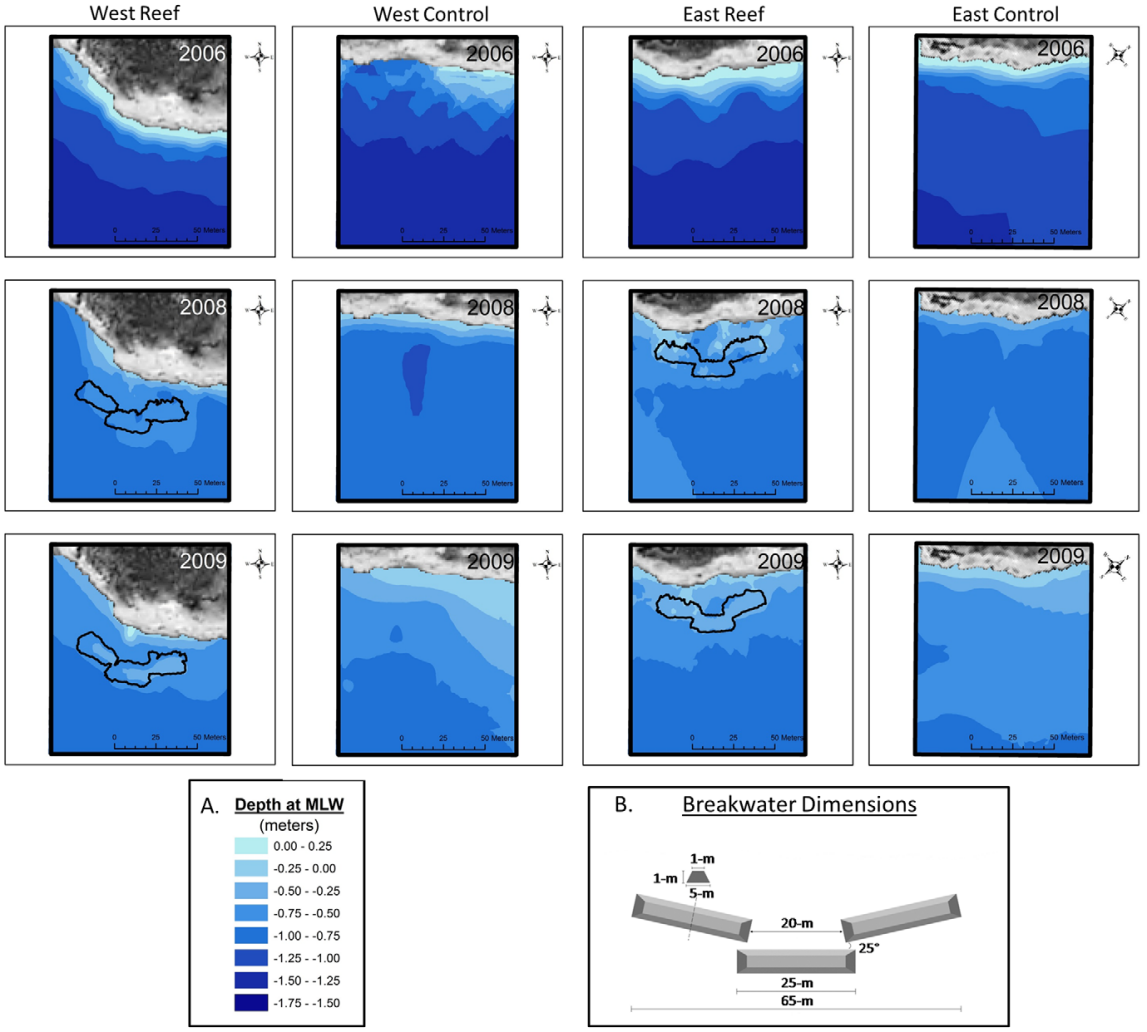
Bathymetry Plots from the Western Experimental Breakwater Reef and Control Treatments at Point aux Pins. The top row of 2006 plots was approximately one year prior to construction. The 2008 and 2009 plots are from one and two years post construction. Depth gradients are shown in inset (A). A schematic of the initial reef shape is depicted in (B). The crest width of each reef was approximately 1-m at MLLW.

The experimental oyster reefs were designed as subtidal wave-attenuating breakwaters, a common coastal engineering approach [8]. Each reef complex was comprised of three 5 m×25 m rectangular-trapezoid sections (Figure 3B). Each section consisted of loose oyster shell, purchased from a local seafood processing plant, placed on a geo-textile fabric to prevent subsidence and secured by a plastic mesh covering (with 1 cm^2^ openings) that was anchored by rebar. The purpose of the mesh covering was to help maintain the vertical relief of breakwaters until adequate recruitment of oysters cemented the loose shell in place. The initial height of each reef was slightly above MLLW (∼1 m), under the assumption that the loose oyster shell would settle below that level and eventually become subtidal. The subtidal design of the reefs allowed for maximum exposure for oyster settlement and increased available substrate for foraging by transient and larger resident fishes, while maximizing potential capacity for wave attenuation.

### Hydrographic Environment

Mean surface water temperatures, recorded by electronic thermometer, and salinity, measured by a refractometer, were recorded during each sampling event. To observe longer term patterns in salinity, we utilized publicly available data recorded by hydrographic monitoring stations located at Cedar Point and Dauphin Island, AL. The Cedar Point station is approximately 17.5 km from Point aux Pins and 4.0 km from Alabama Port site center points. The Dauphin Island station is approximately 25.5 km from Point aux Pins and 11.0 km from Alabama Port site center points. The Cedar Point station has been active since 2008 and the Dauphin Island station since 2003. To consider the effects of wave climate and dominant wind direction and magnitude on our study setting, we reviewed historical and recently published coastal engineering studies [26], [27].

### Shoreline and Bathymetry Change

Vegetation retreat and changes in nearshore depth profiles were monitored to evaluate the effect of the breakwaters on the nearshore setting. Bathymetry surveys were conducted at both sites during preliminary site selection and yearly following construction at Point aux Pins. Bathymetric data was collected using a Ceeducer Pro DGPS system with an integrated depth sounder mounted to a 1 m×2 m platform on pontoons. We surveyed each site manually by walking the pontoon through multiple parallel transects of the reef and control treatments. At each reef treatment, the breakwater reef footprint was delineated using the Ceeducer DGPS to measure reef spreading and consequential reduction in reef height. The width of each reef section was also measured by transect tape at reef construction and the end of the study to measure changes in reef footprint. The data collected by the Ceeducer unit was imported in ESRI's ArcView, corrected for tidal amplitude, and maps depicting depth at mean low water (MLW) were created. To measure the shoreward retreat of emergent vegetation, permanent rebar stakes were installed at 25 m intervals along the 100 m stretch of shoreline at each replicate treatment. Each 6 m rebar stake was driven into the marsh edge so that 1 m remained visible. These shoreline stakes were installed shortly after breakwater construction at both Point aux Pins and Alabama Port and were monitored periodically thereafter. During each survey, marsh retreat was measured as the distance from the rebar stake to the living vegetation line. Mean differences between vegetation retreat rates adjacent to breakwaters and controls were analyzed by repeated-measures analysis of variance (ANOVA). Because of differences in reef creation dates and sampling period, Point aux Pins and Alabama Port were analyzed separately.

### Oyster Recruitment

To assess the value of the breakwater reef complexes for oysters and other sessile invertebrates, we periodically collected quadrat samples. Oyster settlement, growth and survival were quantified using a 0.25 m^2^ quadrat, which was haphazardly placed at three locations on each reef section (n = 9 per replicate reef). The exposed layer of shell within the quadrat was collected and placed in a large container. Juvenile (≤3 cm) and adult oysters (>3 cm) were enumerated and measured in the field, and then returned to the reef. Mortality was quantified by enumerating dead oysters, which had both valves still articulated and were absent of fouling organisms inside the shell. We sampled the breakwater reefs at Point aux Pins in July, August and November 2007, May and October 2008 and June 2009. We sampled the reefs at Alabama Port in March, June and October 2008 and June 2009. For the final sampling period of June 2009, six 0.25 m^2^ quadrats were sampled from each section (n = 18 per replicate reef) to account for the reef spreading and to assure a similar proportion of reef surface area was sampled.

We used univariate one-way ANOVA to test for differences in densities of live juveniles, live adults and dead oysters among sampling events. Point aux Pins and Alabama Port recruitment data were analyzed separately because the independent variable of sampling date was different at each site. Density estimates determined by individual quadrat samples (n = 3 all, except n = 6 for June 2009) for each reef section were averaged. The pooled values from each of the three reef sections for each of the two replicated treatments were used as replicates in a one-factor ANOVA to test the effect of sampling date. These data were tested for normality using the Kolmogorov-Smirnov test and homogeneity of variances using Bartlett's test. To meet the assumptions of ANOVA, all values were log transformed and retested. After transformation, minor violations of normality and equal variances were still present for live adults and dead oysters at both sites. Because the violations from quadrat sampling are generally minor and ANOVA is considered robust to such violation [28], we proceeded with parametric ANOVA. When ANOVA results showed significant differences, we used Tukey's HSD post-hoc test for multiple comparisons.

### Fishes and Mobile Invertebrates

The response of fishes and mobile invertebrates was measured using a combination of gear types to target small and large individuals. Experimental gillnets (2 m×30 m) were used to capture larger species and individuals of coastal finfish species. Sampling occurred twice each month for one year following construction and monthly thereafter through all seasons, but was reduced to every other month during winter. Gillnets were deployed on adjacent sides of each reef or control treatment and perpendicular to shore. Each net was comprised of two 15 m panels (5 cm and 10 cm maximum opening) to broaden the size range and body shape of animals captured. Gillnets were fished for two hours starting one hour prior to sunrise. During winter months, low tides prevented crepuscular sampling so nets were fished for two hours starting one hour prior to sunset. Gillnets were retrieved in the same order they were deployed, and soak time was recorded as the time from when the net was first deployed until the time retrieval began. All specimens captured were placed in labeled bags and returned to the lab where they were identified, measured and their biomass recorded.

To quantify smaller fishes and invertebrates, we seined adjacent to each breakwater reef and control monthly, except every other month during winter. At each treatment, a 6 m wide bag seine with 6.25 mm mesh was towed three times between the treatment and shore. All seine distances were 15 m and terminated into the shore at Point aux Pins or a 4 m wide block net at Alabama Port. All captured mobile invertebrates and fishes were placed in labeled bags and returned to the laboratory where they were identified to the lowest taxonomic level possible, measured and biomass recorded.

To determine the effects of site and treatment on the communities of fishes and invertebrates, we used multivariate and univariate analyses. Differences in community structure between reef and control treatments and between Alabama Port and Point aux Pins sites were tested for each gear type using permutational analysis of variance (PERMANOVA). Multivariate PERMANOVA used Bray-Curtis similarity matrices of log (x+1) transformed abundance data with 4,999 permutations [29]. Logarithmic transformations were applied to reduce the influence of overwhelmingly abundant species. For univariate analyses on gillnet data, PERMANOVA was used to test for site and treatment effects on the total abundance, species richness and abundance of demersal fishes in an approach similar to parametric ANOVA. Univariate PERMANOVA tests were run on Euclidean distances matrices with 4,999 permutations [30]. PERMANOVA was chosen for univariate analyses because it allows for two-factor designs, considers an interaction term and does not assume a normal distribution of errors. The environmental classifications of demersal, pelagic (including benthopelagic, pelagic, and pelagic-neritic) and reef-associated fishes were acquired from FISHBASE [31]. Seine data were analyzed identically to gillnet data analyses as previously stated with the addition of a response variable containing only decapod crustaceans. All multivariate tests and univariate PERMANVOA were run in the software package PRIMER-E v6 [32] with the PERMANOVA extension.

To determine the effects of breakwater reefs on the most common demersal fishes and decapods, we analyzed these taxa separately as they include many economically-important coastal species. We used Wilcoxon signed-rank tests to compare relative abundances of each species (≥1%) between the paired breakwater reef and mudflat control treatments. This approach allowed us to test for overall treatment effects, while controlling for date and site variability through the paired experimental design but ignored their interactive effects. Certain species that were closely related or difficult to distinguish were analyzed as grouped taxa (e.g. *Menidia* sp., *Paralichthys* sp.). For all tests, we considered results of p≤0.05 to be significant. The ANOVA and Wilcoxon tests were run using the R Statistical Platform Version 10.1.1 [33].

## Results

### Hydrographic Environment

**Figure 4:**
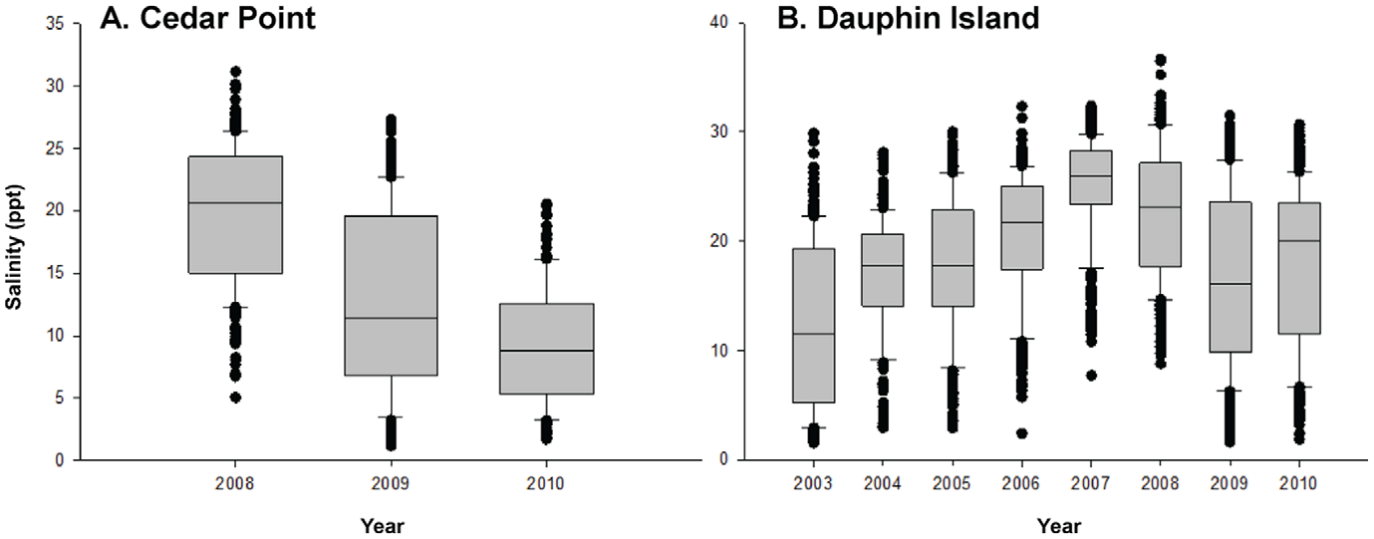
Salinity Ranges Recorded by Hydrographic Monitoring Stations in Coastal Alabama. Box and whisker plots of salinity data recorded by the hydrographic monitoring stations at (A) Cedar Point and (B) Dauphin Island. The Cedar Point Station has been active since 2008 and the Dauphin Island Station since 2003.

At Point aux Pins, mean surface water temperature over all sampling events was 21.4°C (±10.1 SD) measured by digital thermometer, and salinity averaged 23.1 PSU (±8.7 SD) measured by refractometer. Mean water temperature at Alabama Port was 21.8°C (±7.8 SD) and salinity averaged 16.1 PSU (±7.4 SD). Salinity data, shown as box and whisker plots, was acquired from hydrographic monitoring stations at Cedar Point (Figure 4A) and Dauphin Island (Figure 4B) to further investigate the salinity regime over a longer time period. Cedar Point data shows 2008 to have the highest salinity regime of the 2008–2010 years (Figure 4A). The Dauphin Island station shows a similar pattern with 2007 and 2008 having higher salinities than all other years between 2003 and 2010. In addition to higher average salinity, the outliers representing the lowest salinity measurements in 2007 and 2008 are substantially higher the other recent years indicating fewer freshets.

Most wind-driven wave energies along coastal Alabama shorelines are generated by dominant south to southeasterly winds from spring through early fall and north-oriented winds from late fall throughout most of winter [26], [27]. Fetch at Point aux Pins averages approximately 15 km with a longest fetch of 32 km. The erosion rate at this site has potentially increased in recent years after Hurricane Katrina opened a one mile gap termed “Katrina Cut” in Dauphin Island, a protective barrier island located due south of Point aux Pins. The wave climate near Alabama Port is strongly affected by prevalent southeast winds, as well as the wakes of ships utilizing the Mobile shipping channel less than ten kilometers to the East. At Alabama Port, average fetch is approximately 21 km with a longest fetch of 34 km. For more detailed discussion of wind and wave climates, erosion, sediment sizes, Keddy exposure values and Knutson et al.'s vegetation success scores, refer to Roland and Douglass (2005) [26].

### Shoreline and Bathymetry Changes

**Figure 5:**
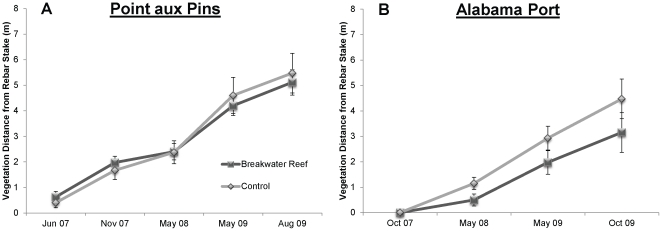
Shoreline Vegetation Retreat. Mean retreat (± SE) of living vegetation shoreward of each treatment at (A) Point aux Pins and (B) Alabama Port.

Changes in the nearshore and shoreline environments of reef and control sites were observed from measuring vegetation retreat and bathymetric surveys. Bathymetric surveys at Point aux Pins found that, in addition to a general trend of decreasing depth, areas inshore of breakwater reefs appeared to gain more sediments than areas inshore of control plots (Figure 3). The footprint of East and West breakwaters expanded approximately 300% over the course of the study, and reef crest height was reduced from approximately 1 m to 0.3 m. The living vegetation line at Point aux Pins retreated nearly 6 m on average in slightly over two years (Figure 5A). Repeated measures ANOVA found no differences in the vegetation retreat rates between treatments, a strong effect of time and no interaction between the two factors (Table S1). At Alabama Port, breakwater reefs mitigated vegetation retreat by more than 40% over two years (Figure 5B). Repeated measures ANOVA found a marginally-significant treatment effect (p = 0.089) and a strong effect of time with no interaction (Table S1).

### Oyster Recruitment

**Figure 6:**
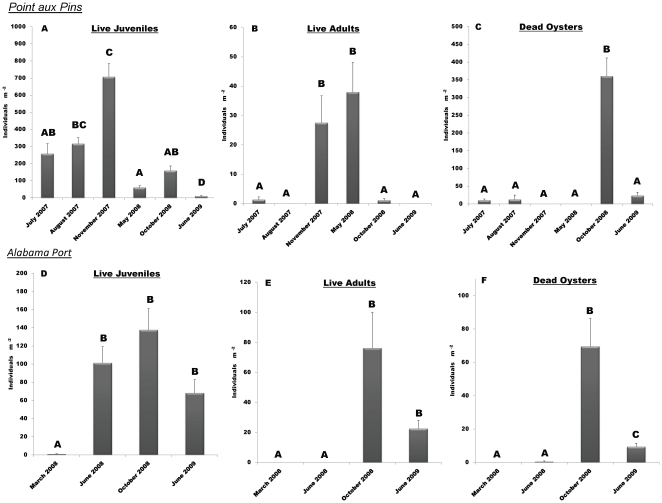
Oyster Recruitment and Survival. Mean oyster densities (+SE) of live juvenile, live adult and dead oysters at Point aux Pins (A–C) and Alabama Port (D–F). Different letters indicate statistical differences (p<0.05) from Tukey's HSD post-hoc tests.

Point aux Pins reefs were constructed in May 2007 and first sampled for oyster recruitment the following July. Densities of juvenile oysters continually increased until peaking at greater than 700 oysters m^−2^ in November 2007, but were much lower the following year with ranges between 50 and 150 m^−2^ (F_5,30_ = 28.15, p≤0.001, Figure 6A). Adult oysters were found in highest densities during November 2007 and May 2008 sampling with approximately 35 oysters m^−2^ (F_5,30_ = 38.29, p≤0.001, Figure 6B). The highest mortality was observed during the October 2008 sampling event (F_5,30_ = 22.492, p≤0.001, Figure 6C) and 88% of measured dead oysters were juveniles (≤3 cm).

Alabama Port reefs were constructed in October 2007 and were first sampled in March 2008. Live juveniles densities at Alabama Port were between 70 and 140 m^−2^ in the last three sampling events and higher than the first sampling event in March 2008 (F_3,20_ = 47.40, p≤0.001, Figure 6D). Adult oysters were observed first and at a maximum in October 2008 (∼75 oysters m^−2^) and found in lower densities in June 2009 (∼20 oysters m^−2^) (F_3,20_ = 18.82, p≤0.001, Figure 6E), although October and June were not significantly different. The first and highest mortality (∼70 oysters m^−2^) was recorded in October 2008 (F_3,20_ = 114.29, p≤0.001, Figure 6F), and juvenile oysters accounted for 80% of the total dead.

### Fishes and Mobile Invertebrates

Gillnet and seine sampling near breakwater reefs and controls captured a diverse assemblage of fishes and mobile macro-invertebrates. From the use of multiple gears, over 100 species of fish and invertebrates were collected during the 30 month sampling period. Gillnet sampling collected nearly 8,000 individuals of 45 different species in 5 cm mesh panels while larger 10 cm panels captured over 1,500 individuals of 44 different species. Seines captured 71,640 individuals that represented 88 species or grouped taxa. Demersal fishes appeared to be the most broadly enhanced by the oyster reef structure when the overall percent difference in CPUE between oyster reefs and mudflat controls was calculated across both sites and all sampling events (Table S2). The dominant pelagic and reef-associated species did not appear strongly affected by oyster reef presence. Of the twelve species that comprised at least 1% of the 5 cm gillnet catch, six were categorized as demersal species. Four of these six demersal taxa were more abundant on breakwater reefs than controls. Spotted seatrout were 38% more abundant near breakwater reefs, and displayed the strongest trend of enhancement among 5 cm captured fishes. Twenty species comprised at least one percent of the 10 cm gillnet catch, and eleven of these were demersal fishes. Fourteen of the twenty species were captured more often near breakwater reefs than controls. Nine species or grouped taxa comprised at least one percent of seine catches, seven of which were more frequently captured near breakwater reefs. Included in these seven were three demersal fishes and three decapod crustaceans.

We used multivariate PERMANOVA to test for differences in the community structure between breakwater reef and control treatments. PERMANOVA tests on 5 cm gillnet catches found that site and the site-treatment interaction were both significant factors (Table S3). There were no community-level differences between our breakwater and control treatments with 5 cm captured fishes. The communities of larger fishes captured by 10 cm gillnets differed significantly by site, treatment and the interaction of the two factors (Table S3). The community structure of smaller and juvenile fishes and mobile invertebrates captured by seines were different between sites and treatments, with no interaction between the two factors (Table S3).

**Figure 7:**
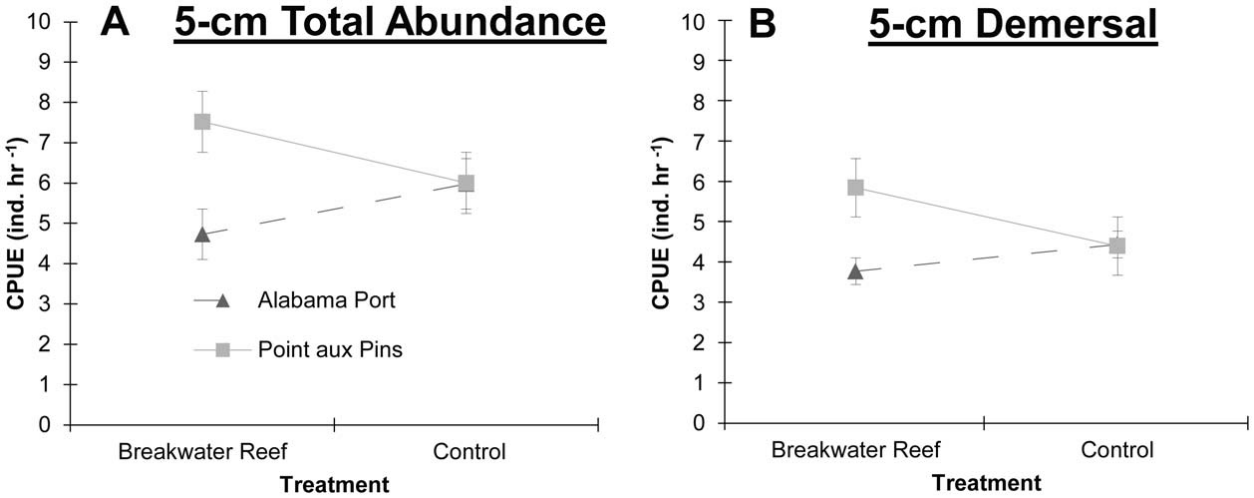
Relative Demersal Fish and Decapod Crustacean Abundance. Mean ±1 SE CPUE of (A) demersal fishes separated by collection method and (B) decapod crustaceans collected by seines near breakwater reefs and controls. Significant differences at P≤0.05 from univariate PERMANOA tests are indicated by asterisks.

**Figure 8:**
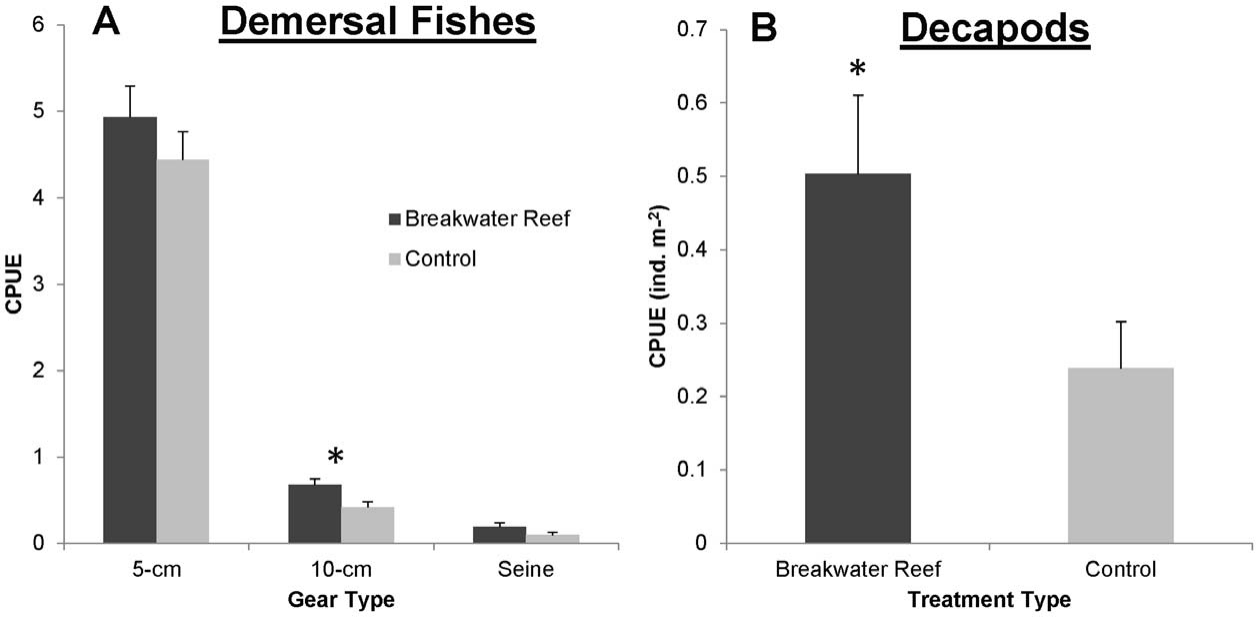
Total Abundance and Demersal Fish Abundance Separated by Site. Mean+1 SE catch per unit effort of (A) total fish and invertebrate abundance and (B) demersal fish abundance collected by 5 cm gillnets. CPUE is presented as the total individuals captured for each hour of soak time.

We used univariate PERMANOVA tests on total abundance, species richness and demersal and decapod abundances to detect differences between breakwater reef and control treatments and between Alabama Port and Point aux Pins. For 5 cm total abundance, a significant interaction between site and treatment was observed (Table S4) because total abundance was higher near breakwaters at Point aux Pins but higher near controls at Alabama Port (Figure 7A). For 10 cm gillnet catch, total abundance was higher adjacent to oyster reefs than controls (Table S4). For both 10 cm and seine data, abundances were significantly higher at Point aux Pins than Alabama Port, and no interaction was observed between site and treatment (Table S4). The PERMANOVA tests on species richness found significant differences between sites across all gear types, between treatments only for 10 cm catches and no significant interactions (Table S4). For 10 cm catches, species richness was significantly higher near reefs than controls (Table S4) and higher at Point aux Pins than Alabama Port. Demersal fishes showed no differences between reef and mudflat treatments for 5-cm catches (Table S3), but again there was a significant interaction between site and treatment(Figure 7B). For 10 cm, demersal fishes were more abundant near breakwater oyster reefs (Figure 8A) and higher at Point aux Pins. From seine catches, demersal fish abundance showed no differences, but decapod crustacean abundance was higher near reefs than mudflat controls (Table S4 and Figure 8B).

**Figure 9:**
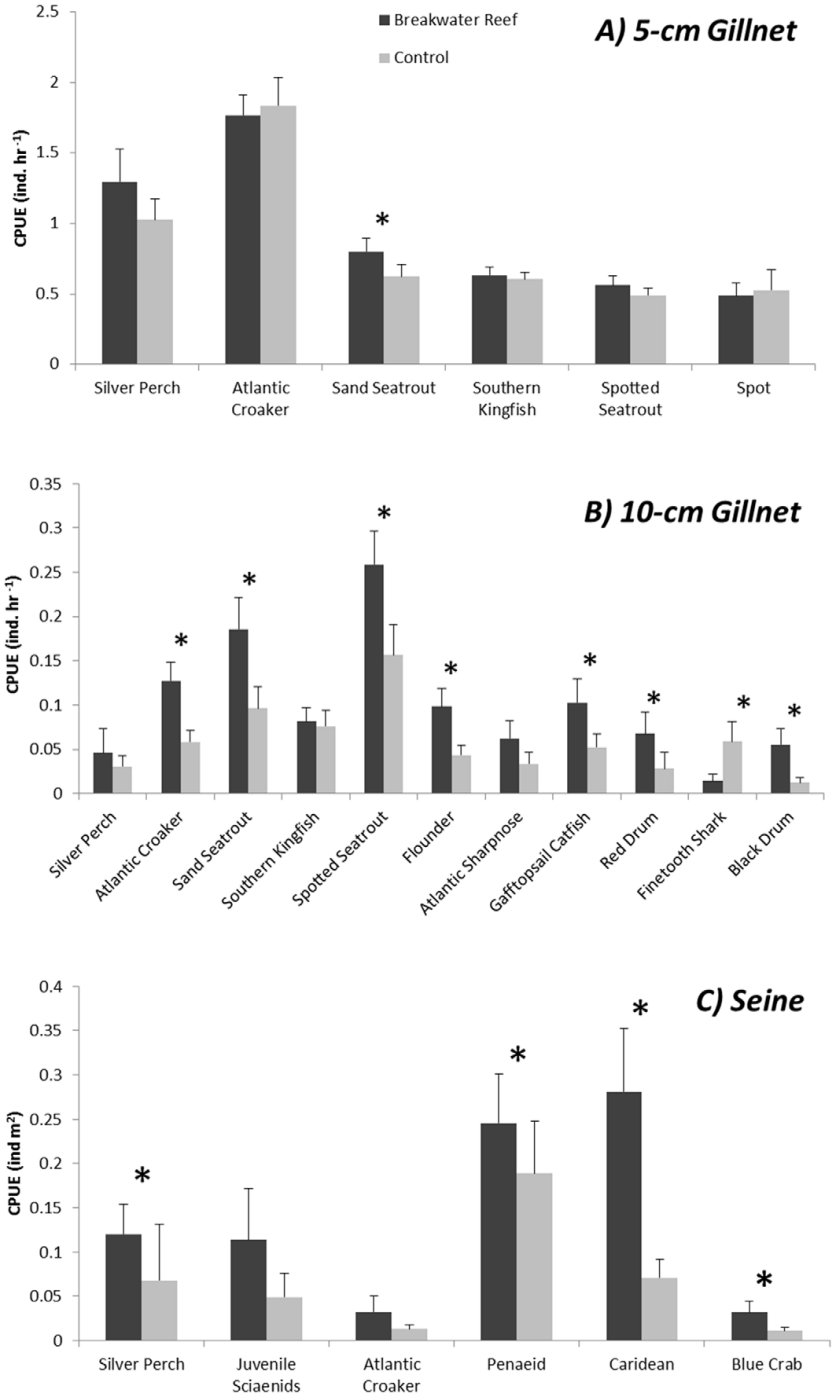
Relative Abundance of Dominant Demersal Fish and Decapod Taxa. Mean+1 SE CPUE of dominant demersal and decapod species or grouped taxa between treatments. Significant differences at P≤0.05 from Wilcoxon signed rank tests comparing paired breakwater reef and control treatments.

The relative abundance of each demersal fish and decapod species (≥1%) between breakwater and control treatments was tested using paired Wilcoxon signed-rank tests. For 5 cm gillnet samples, six demersal species contributed ≥1% of the total catch (Table S5). Of those, only sand seatrout abundance was significantly enhanced by breakwater reefs (Figure 9A). Silver perch, spotted seatrout and southern kingfish showed positive trends of enhancement, but not statistically significant. Eleven demersal fishes were analyzed from the 10 cm catches, seven of which were significantly enhanced by reefs including sand seatrout, spotted seatrout, red drum and black drum (Table S5 and Figure 9B). Only finetooth shark abundance in 10 cm gillnets was significantly greater on controls than breakwater reef treatments. Seine samples had nine species or taxa that comprised ≥1% of the total catch, including three demersal fish species and three decapods. Of the demersal fishes, which were silver perch, Atlantic croaker and juvenile sciaenids, only silver perch showed a significant difference and were more common near breakwater reefs (Figure 9C). All three decapods, caridean shrimp, penaeid shrimp and blue crabs were present in significantly higher densities near breakwater reefs.

## Discussion

Our study found that breakwater reefs constructed of loose oyster shell provided substrate for oyster recruitment and harbored a more diverse community of fishes and mobile invertebrates than control areas without reefs. This habitat enhancement is uncommon among shoreline protection schemes and could be a vast improvement over traditional armoring techniques, many of which have detrimental impacts on nearshore species [16]. While our experimental breakwaters were an “ecology-first” approach and were successful in creating valuable habitat, they did not provide the amount of protection that could be offered by well-engineered methodologies. This shortcoming highlights the need for coastal protection philosophies that balance ecology and engineering. However, an approach similar to ours could serve as an immediate solution to the habitat losses experienced along many sheltered coasts. In these settings, breakwater oyster reefs that were installed seaward of already armored shorelines could mitigate losses of fish and shellfish habitat.

Roland and Douglass (2005) found that many stretches of Alabama's shoreline are faced with wave energies well above critical limits where vegetation can naturally persist and proposed breakwaters as a potential mechanism to reduce wave energies [26]. The wave-attenuating capacity of the breakwaters in our study was compromised because the loose shell reefs expanded and flattened prior to the cementing together that could result from oyster settlement and survival. The mesh covering used in our study to maintain the breakwater reefs' integrity was not rigid enough to withstand the wave energy of our sites, but an improvement in this aspect of the breakwater design could allow for better shoreline protection and less disturbance of the reef. To mitigate reef spreading and flattening, we suggest the introduction of a more rigid structure as a temporary backbone which would deteriorate or could be removed after reef cementing occurred.

At both Alabama Port and Point aux Pins, we documented oyster recruitment and survival to reproductive size, but substantial mortality limited reef cementing and success. The high mortality recorded at both sites during October 2008 sampling appeared to be caused by predation or physical disturbance, such as wave energy. During this sampling period, very few exposed oysters were observed to be alive. In contrast, nearly all live oysters observed were found sheltered inside of dead, but still hinged oyster shells. This suggests that it is unlikely disease was the cause of mortality, since structurally-protected oysters would have no reprieve. Another factor that frequently affects oyster survival is reef height as tall reefs escape the poor water quality sometimes found near the sediment [34]. As previously discussed, the vertical relief of our reefs did decline over time, but again it is unlikely that sheltered oysters would survive if water quality caused the observed mortality. Physical disturbance could have caused many of the oyster shells that were on the surface and available for settlement to be buried under other shells, also explaining the lowered densities of live oysters. Predation is likely the most plausible explanation for the differential mortality between sheltered and exposed oysters. We frequently observed black drum, southern oyster drills (*Stramonita haemastoma*) and several species of crabs near the reefs. Stomach content analysis of the black drum collected in gillnets usually found oyster shell remains and dead oysters often showed signs of predation (S Scyphers, Pers. Observ.). A recent mark and recapture study of subtidal oyster reefs in coastal Alabama waters also documented drills as the most prevalent cause of mortality due to visible scarring on dead spat shells [35]. The high salinities and absence of freshets observed during the drought conditions 2007 and 2008 were likely beneficial for the oyster drill predators which thrive in higher salinity conditions [36], [37].

The communities of fishes and mobile invertebrates that benefit from oyster reefs have been well-described, but very few studies have examined the enhancement from oyster reefs designed for protecting shorelines. The elevated species richness and densities that we observed during our study concur with most literature describing oyster reef habitats [2]. From our seines, we found blue crabs, penaeid and caridean shrimp, and juvenile silver perch were more abundant near oyster reefs than mudflat controls. Higher blue crab densities near reefs were likely due to the refuge value, as their recruitment and survival is largely augmented by structured habitats [38]. Blue crabs support an important commercial fishery throughout Gulf and Atlantic estuaries and, along with caridean and penaeid shrimp, are commonly found in the diets of several of the larger fishes. From our 10 cm gillnet sampling, we found that spotted seatrout, drum and flounder were substantially enhanced by oyster reefs. The paradigm of abundance, biomass and species richness being higher in structured areas and further increasing with habitat complexity is a pattern observed in nearly all nearshore ecosystems [20], [39]–[41], but the relative importance of food versus refuge within structured habitats remains unresolved [42], [43].

Landscape attributes, such as adjacent habitats or bathymetric features, commonly influence community composition [44]–[46] and are probably responsible for the interaction between site and treatment for the total abundance and demersal abundance of 5 cm gillnet catches. The interaction was driven by demersal fishes (Figure 7) and these catches were dominated by Atlantic croaker and silver perch, both which are recognized to predominately feed in non-structured habitats [47]. Geraldi et al. (2009) found very little evidence of enhancement by oyster reefs restored in marsh tidal creeks and concluded that the area was not limited by complex structure and therefore the addition of oyster shell was functionally redundant. Grabowski et al. 2005 concluded that small or few reefs may not measurably enhance transient predators. Interestingly, the broad enhancement we observed occurred in a similar setting with each reef located near structurally-complex saltmarsh habitat and of moderate size (∼225 m^2^).

It has proven quite challenging to predict the ecosystem services to be expected from restoring reefs at different scales or in different settings [34], [41], [42]. Ecosystem services provided by shallow marine habitats have received considerable attention from natural and social scientists seeking to quantify and predict potential benefits from protection or restoration [5], [48], [49]. Historically, most of these studies have focused on wetlands, seagrass meadows, coral reefs and mangroves [5], [50], [51], all habitats that receive considerable protection because of their productivity. Oyster reefs also provide important ecosystem services [18], but are more challenging to protect and manage because they are an exploited fishery [2]. A long history of excessive and destructive harvesting coupled with natural stressors like disease and storms have left shellfish populations in global demise [52]–[54]. Most large or landscape scale oyster reef restoration efforts have primarily targeted the re-establishment of harvestable oysters, many of which failed to achieve previous population levels. Some recent studies have detailed shortcomings of oyster restoration and cast serious doubts on the ability to achieve restoration success in subtidal and often large-scale efforts [55]. However, other recent studies have documented restored reefs that have persisted over decades [56] and on unrivaled spatial scales [57]. Attempts to quantify the economic benefits from restoring oyster reefs are very recent and forthcoming and could provide more support for protecting and restoring oyster reefs for the goods and services they provide [58], [59].

Awareness of the detrimental impacts of shoreline armoring has increased in recent years, but movement towards more ecologically-responsible methods has been limited by the lack of cost-effective alternatives. “Living shoreline” approaches, including breakwater reefs, that protect coastal uplands could provide a more ecologically-responsible alternative to traditional armoring and not only mitigate coastal erosion, but also enhance certain economically-valuable fish stocks. However, as our study demonstrated, efforts to sustainably and responsibly protect coastal shoreline habitats must balance both engineering and ecology.

## Supporting Information

Table S1 **Results of Repeated-Measures ANOVA on Vegetation Retreat.**
(DOCX)

Table S2 **Relative Abundance of the Most Abundant Fishes and Mobile Invertebrates.**
(DOCX)

Table S3 **Results from Multivariate PERMANOVA Tests.**
(DOCX)

Table S4 **Results from Univariate PERMANOVA Tests.**
(DOCX)

Table S5 **Results from Wilcoxon Signed-Rank Tests on Single Species or Grouped Taxa.**
(DOCX)
