# Supplementary material for: Oyster Reefs as Natural Breakwaters Mitigate Shoreline Loss and Facilitate Fisheries
Source: PLoS One. 2011 Aug 5;6(8):e22396. doi: 10.1371/journal.pone.0022396 (PMC3151262; doi:10.1371/journal.pone.0022396)
Supplement: Table S1 — Results of Repeated-Measures ANOVA on Vegetation Retreat. (DOCX) [file pone.0022396.s001.docx]

Table S1. Results of repeated-measures ANOVA tests on vegetation retreat between reef and control treatments at each site.
