# Supplementary material for: Oyster Reefs as Natural Breakwaters Mitigate Shoreline Loss and Facilitate Fisheries
Source: PLoS One. 2011 Aug 5;6(8):e22396. doi: 10.1371/journal.pone.0022396 (PMC3151262; doi:10.1371/journal.pone.0022396)
Supplement: Table S2 — Relative Abundance of the Most Abundant Fishes and Mobile Invertebrates. (DOCX) [file pone.0022396.s002.docx]

Table S2. Relative abundance of the most prevalent fishes and mobile invertebrates (≥1%) between experimental breakwater and control treatments. Positive values indicate species that were more abundant near breakwater reefs and negative values indicate higher abundance near controls. Environment classifications are demersal (D), pelagic (P), reef-associated (RA) fishes, and decapods crustaceans (DE). Demersal fishes and decapods are highlighted in gray and were analyzed by Wilcoxon signed-rank tests.
