# Supplementary material for: Oyster Reefs as Natural Breakwaters Mitigate Shoreline Loss and Facilitate Fisheries
Source: PLoS One. 2011 Aug 5;6(8):e22396. doi: 10.1371/journal.pone.0022396 (PMC3151262; doi:10.1371/journal.pone.0022396)
Supplement: Table S3 — Results from Multivariate PERMANOVA Tests. (DOCX) [file pone.0022396.s003.docx]

Table S3. Results from multivariate PERMANOVA using Bray-Curtis similarity values to test for site and treatment effects on community structure. The test statistic (F*) is a pseudo-F value and the probability values (P^†^) are computed by the PERMANOVA routine with 4,999 permutations on gillnet (n=256) and seine (n=176) catches.
