# Supplementary material for: Oyster Reefs as Natural Breakwaters Mitigate Shoreline Loss and Facilitate Fisheries
Source: PLoS One. 2011 Aug 5;6(8):e22396. doi: 10.1371/journal.pone.0022396 (PMC3151262; doi:10.1371/journal.pone.0022396)
Supplement: Table S4 — Results from Univariate PERMANOVA Tests. (DOCX) [file pone.0022396.s004.docx]

Table S4. Results from univariate PERMANOVA on Euclidean distances to test for site (Alabama Port or Point aux Pins) or treatment (Breakwater Reef or Control) differences on total abundance, species richness, demersal abundance, and decapod abundance for each gear type. Footnotes: The test statistic (F*) is a pseudo-F value and the probability value (P^†^) are computed by the PERMANOVA routine with 4,999 permutations on gillnet (n=256) and seine (n=176) catches.
