# Supplementary material for: Oyster Reefs as Natural Breakwaters Mitigate Shoreline Loss and Facilitate Fisheries
Source: PLoS One. 2011 Aug 5;6(8):e22396. doi: 10.1371/journal.pone.0022396 (PMC3151262; doi:10.1371/journal.pone.0022396)
Supplement: Table S5 — Results from Wilcoxon Signed-Rank Tests on Single Species or Grouped Taxa. (DOCX) [file pone.0022396.s005.docx]

Table S5. Test results for demersal fishes from gillnet and seine samples and decapods that comprised one percent or greater of the total catch for each gear type. Wilcoxon signed-rank tests were used to compare relative abundances between breakwater reefs and controls on gillnet (n=128) and seine (n=88) catches. P-values from Wilcoxon signed rank tests comparing paired breakwater reef and control treatments are shown and significant differences at P ≤ 0.05 indicated by asterisk. These results are depicted in Figure 9A-C.
